# Gastric cancer diagnosis using hyperspectral imaging with principal component analysis and spectral angle mapper

**DOI:** 10.1117/1.JBO.25.6.066005

**Published:** 2020-06-27

**Authors:** Ningliang Liu, Yaxiong Guo, Houmin Jiang, Weisong Yi

**Affiliations:** a Huazhong Agricultural University, College of Science, Wuhan, China; b People’s Hospital of Huangpi District, Wuhan, China

**Keywords:** gastric cancer, hyperspectral imaging, diagnosis, principal component analysis, spectral angle mapper

## Abstract

**Significance**: Hyperspectral imaging (HSI) is an emerging optical technique that has a double function of spectroscopy and imaging.

**Aim**: Near-infrared hyperspectral imaging (NIR-HSI) (900 to 1700 nm) with the help of chemometrics was investigated for gastric cancer diagnosis.

**Approach**: Mean spectra and standard deviation of normal and cancerous pixels were extracted. Principal component analysis (PCA) was used to compress the dimension of hypercube data and select the optimal wavelengths. Moreover, spectral angle mapper (SAM) was utilized as chemometrics to discriminate gastric cancer from normal.

**Results**: Major spectral difference of cancerous and normal gastric tissue was observed around 975, 1215, and 1450 nm by comparison. A total of six wavelengths (i.e., 975, 1075, 1215, 1275, 1390, and 1450 nm) were then selected as optimal wavelengths by PCA. The accuracy using SAM is up to 90% according to hematoxylin–eosin results.

**Conclusions**: These results suggest that NIR-HSI has the potential as a cutting-edge optical diagnostic technique for gastric cancer diagnosis with suitable chemometrics.

## 1 Introduction

Gastric cancer is one of the most serious illnesses in the world because of its high morbidity and mortality.^1^ Currently, early detection and localization of gastric cancer sites are critical to decrease the mortality. However, it is a big challenge for clinicians who routinely utilize conventional white-light reflectance endoscope to accurately identify and localize early dysplasia, carcinoma *in situ*, and flat mucosal cancers in the stomach.^2^ The serum markers fail to diagnose or screen gastric cancer with sufficient sensitivity and specificity despite being helpful for monitoring response to therapy and detecting cancer recurrence. Although the radiological diagnostic tests, ionizing or nonionizing radiology, are proved to be effective for gastric cancer diagnosis, a positive result requires further histopathology examination [hematoxylin–eosin (H&E)], which could finally decide when and how to operate on the tumor. The result with H&E approach is invasive, time-consuming, and impractical as a routine screening tool for high-risk patients, and is also subjective based on the pathologist’s expertise.^3^ Yet accurate identification of the tumor margins is of remarkable clinical importance, in particular for the diffuse type of gastric cancer where the boundaries of the lesion can be indistinct and nests of cancerous cells may be found at a distance from the visible tumor margin. As an emerging spectroscopy and imaging modality for medical applications, hyperspectral imaging (HSI) offers great potential for noninvasive disease diagnosis and surgical guidance.^4^^,^^5^

For gastric cancer, Akbari et al.^6^ utilized infrared (IR)-HSI (1000 to 2500 nm) to investigate gastric cancer with the help of chemometrics, such as spectral standard deviation, support vector machine, integral method, normalized cancer index, and laid the ground for gastric cancer discrimination. Kiyotoki et al. developed the HSI system (400 to 800 nm) to obtain HSI comprising 72 spectral bands with a spatial dimension of 640×480  pixels. They established a diagnostic algorithm to detect gastric cancer using the cutoﬀ point method at the 726-nm wavelength based on spectral reflectance obtained from normal mucosa and tumors.^7^ The sensitivity, specificity, and accuracy rates of the algorithm’s diagnostic capability were 78.8%, 92.5%, and 85.6%, respectively. Goto et al.^8^ examined the difference in the spectral reflectance of gastric tumors and normal mucosa recorded with the same HSI as Kiyotoki et al. A wavelength of 770 nm and a cutoff value of 1/4 the corrected spectral reflectance were selected as the respective optimal wavelength and cutoff values for differentiating tumors from normal mucosa to establish a diagnostic algorithm. The rates of sensitivity, specificity, and accuracy of the algorithm’s diagnostic capability were 71%, 98%, and 85%, respectively. In 2019, Baltussen et al. developed the HSI system (400 to 1700 nm) to distinguished normal fatty tissue, healthy colorectal mucosa, and adenocarcinoma to provide more diagnostic information during endoscopic procedures. After feature reduction, a quadratic classifier and support vector machine were used to distinguish the three tissue types from tissue samples of 32 patients. The tissue-level accuracy and patient-level accuracy were 88% and 93%, respectively.^9^ To some extent, these results demonstrated the feasibility of HSI with chemometrics to discriminate gastric cancer from normal tissue with relatively high sensitivity, specificity, and accuracy. Although the classiﬁcation method employed to distinguish the diﬀerent types of samples *ex vivo* was quite basic, these studies revealed promising results in the use of HSI as a diagnostic tool for gastric cancer. On the other hand, the diagnostic capability of HSI with different algorithms was remarkably different, cancer discrimination algorithms might be optimized and improved. Mostly *in vivo* research work using HSI has focused on easily attachable tissues, such as brain, head and neck, breast, skin, oral, and laryngeal.^4^^,^^5^ For the deeper gastrointestinal system, the first research on colorectal tumors *in vivo* was performed by Han et al.^10^ They developed an HSI endoscopic system (405 to 665 nm) that was based on a motorized filter wheel and capable of obtaining 27 different bands, to discriminate between malignant colorectal tumors and normal colonic mucosa in human patients. The wavelength selection algorithm based on the recursive divergence method was used to identify the most relevant wavelengths in the spectral range employed. The sensitivity and specificity results achieved in this study reach up to 96% and 91%, respectively, using all the available bands. The results demonstrated that HSI has the potential to provide an innovative tool for image-guided surgery.

Our team has investigated the feasibility of near-IR HSI (900 to 1700 nm) (NIR-HSI) for gastric cancer detection with minimum noise fraction transform and cancer target detection algorithms.^11^ Chemometric pattern recognition approaches including nonsupervised and supervised are frequently applied to large databases of spectra to extract relevant biochemical information related to disease and to convert that information into a predicted diagnosis.^12^^,^^13^ When classification algorithms are applied to medical HSI data, these algorithms face two main challenges: the high dimensionality and the limited number of samples. However, these challenges are not necessarily current in other HSI domains but are more prevalent in medical HSI because of substantial interpatient spectral variability.^4^ Here, this research investigates the feasibility and effectiveness of gastric cancer diagnosis using NIR-HSI with chemometrics mainly including principal component analysis (PCA) and spectral angle mapper (SAM). Nonsupervised PCA is the most widely used dimensionality reduction method for medical hyperspectral dataset analysis.^3^ PCA could remarkably reduce the dimensionality of a hyperspectral image and select the optimal wavelengths as later input endmember.^14^ SAM is a supervised image classification method that allows rapid mapping of similarity degree between image spectra and reference spectra with pixel level.^15^ Because of the convex–concave of the stomach surface and nonuniformity of the illumination device, SAM is relatively effective when used on calibrated reflectance data, because it is insensitive to illumination and albedo effects.

## 2 Materials and Methods

### 2.1 NIR-HSI System

The NIR-HSI system, Hyperspec NIR XS-100 (Headwall Photonics, Fitchburg, Massachusetts), consists of a 14-bit InGaAs charge-coupled device line array detector, a push broom imaging spectrograph, a standard f/2.0 C-mount lens, an illumination unit of two 180-W tungsten halogen lamps with a slit (18 mm in length, 25  μm in width), and a sample transport mechanism. The detector captures reflected light from samples line by line in the NIR spectral range from 900 to 1700 nm, in which there are totally 168 bands in the case of ∼5-nm intervals. The spatial resolution of the system is about 0.8 mm/pixel. The image acquisition software is Hyperspec^®^ (Headwall Photonics, Fitchburg, Massachusetts).

### 2.2 Image Acquisition

Gastric cancer specimens were collected immediately from randomly selected patients who underwent partial gastric resection for tumor removal at People’s Hospital of Huangpi District, Wuhan, China. A total of 29 samples, 17 males and 12 females, the mean age of the patients was 54 years with the oldest 80 years and the youngest 26 years. All specimens, mucosa surface upward, were kept on a black plastic plane. Surface moisture was wiped by absorbent paper before image acquisition at room temperature. To acquire hyperspectral images, the transport mechanism was moved at a constant speed of 2 mm/s. After measuring and marking the tissue surface, these specimens then were sent to H&E. The study was approved by the local ethics committee, and informed consent for use of samples was obtained from all patients.

### 2.3 Image Processing

Image processing, spectral extraction and analysis were carried out by the Environment for Visualizing Images (ENVI 4.7) software (Research Systems Inc., Boulder, Colorado). In addition, the scientific graphing software Origin 8.0 (OriginLab, Northampton, Massachusetts) was also used for spectral analysis.

To eliminate the influence of the dark current and the nonuniformity of the illumination device, the raw hyperspectral image should be calibrated and normalized. A standard reference white panel was placed in the scene of imaging and its data were utilized as the white reference. The reflectance from the white panel provides an estimate of the incident light on the tissues at each wavelength. The dark current was captured by turning off the light source along with completely covering the lens of the detector with its opaque cap and recording the detector response. Then the data were normalized to find a relative reflectance using the equation.^6^

After calibration and normalization, a mask was created to remove background noise at 1181 nm. A mask image is a binary one that consists of values of 0 and 1. When a mask image is used in a processing function, ENVI includes the areas with values of 1 and ignores the masked 0 values in the calculations. Only the masked image was subjected to further data analysis.

### 2.4 Spectrum Extraction and Analysis

The H&E result as the “gold standard” was applied to extract characteristic spectra of cancerous and normal tissues. The region of interest (ROI) of gastric cancer was manually selected in a zoom window containing the cancerous areas using the circle drawing mode provided by ENVI ROI tool. A small circle (4 pixels) was first drawn within a selected lesion, and the circle was then grown to merge the neighboring diseased pixels using a specified standard deviation (20%) away from the mean of the drawn region. The value of the standard deviation multiplier was determined by visual inspection of the increased ROI in such a way that all the pixels in the grown area were cancerous and the edge pixels were also avoided at the same time. This “grow” ROI selection method is especially effective for selecting the diseased areas with irregular shapes.^16^ The cancerous mean spectrum was calculated among all diseased pixels. The mean spectrum of normal tissue was extracted by the same flowchart and approach.

### 2.5 Cancer Diagnosis

Since hyperspectral images have large amounts of hypercube data containing spectral and spatial information, dimensionality reduction is one of the most important steps during the spectral analysis. To reduce the dimension of the hyperspectral image, PCA was employed to extract a set of orthogonal principal components (PCs) comprising scores and loadings that account for the maximum variance in spectral datasets.^17^ The scores of PCA represent the weighted sums of the original variables without significant loss of useful information, and the loadings can be used to identify important variables that are responsible for the specific features appeared in the corresponding scores.^18^ The optimal wavelengths were selected based on the maximum and minimum loadings of the PCs with highest weights, which contributed most spectral variance between cancerous and normal.

The selected optimal wavelengths were used to replace the full wavelengths for diagnosis with SAM. SAM is a supervised classification method that allows rapid mapping of the degree of similarity between image spectra and reference spectra. Smaller angles represent closer matches to the reference spectra.^15^ This simple classification tool is often used as a first approach to the hyperspectral data and reliable when images have brightness shifts and other spectral artifacts present compare to other classification algorithms. The appropriate reference spectra were selected from five training samples according to H&E to train SAM classifier. Then, the trained SAM classifier was used to diagnose 24 new samples for model verification, and histopathological results served as the “gold standard” for assessment of the diagnostic effect of HSI technique.

## 3 Results and Discussion

### 3.1 Hyperspectral Images

**Fig. 1:**
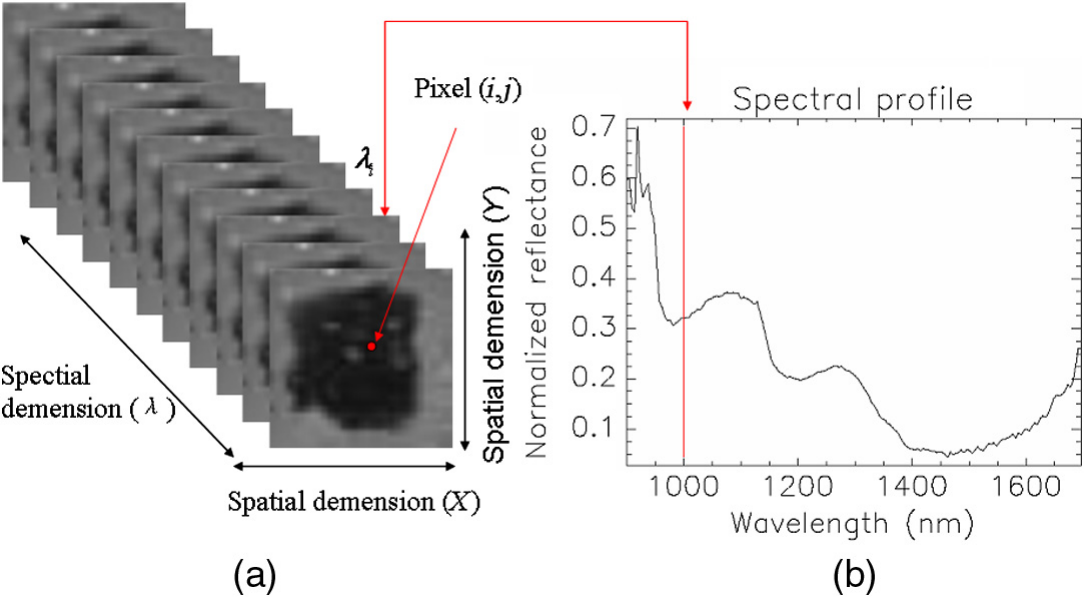
HSI hypercube diagram. (a) Hypercube data including spectral and spatial dimensions. (b) Reflectance spectrum of the pixel (i,j) (red dot).

A hyperspectral image, known as a hypercube, contains three-dimensional block data that provide spatial information along with spectral information for each pixel in each image, as shown in Fig. 1(a). A hyperspectral image is made up of 168 contiguous wavebands for each pixel. Each pixel in the hyperspectral image has a sequence of intensities in different wavelengths, as shown in Fig. 1(b), which constructs the spectral signature of that pixel. The resulting spectrum acts like a fingerprint, reflecting the composition of a certain pixel. The difference in spectral signature between the cancerous and the normal tissues can be distinguished. Hyperspectral images allow for the visualization of biochemical constituents of each pixel, separated into particular areas since similar spectral properties have similar chemical composition.^18^

### 3.2 Characteristic Spectra

**Fig. 2:**
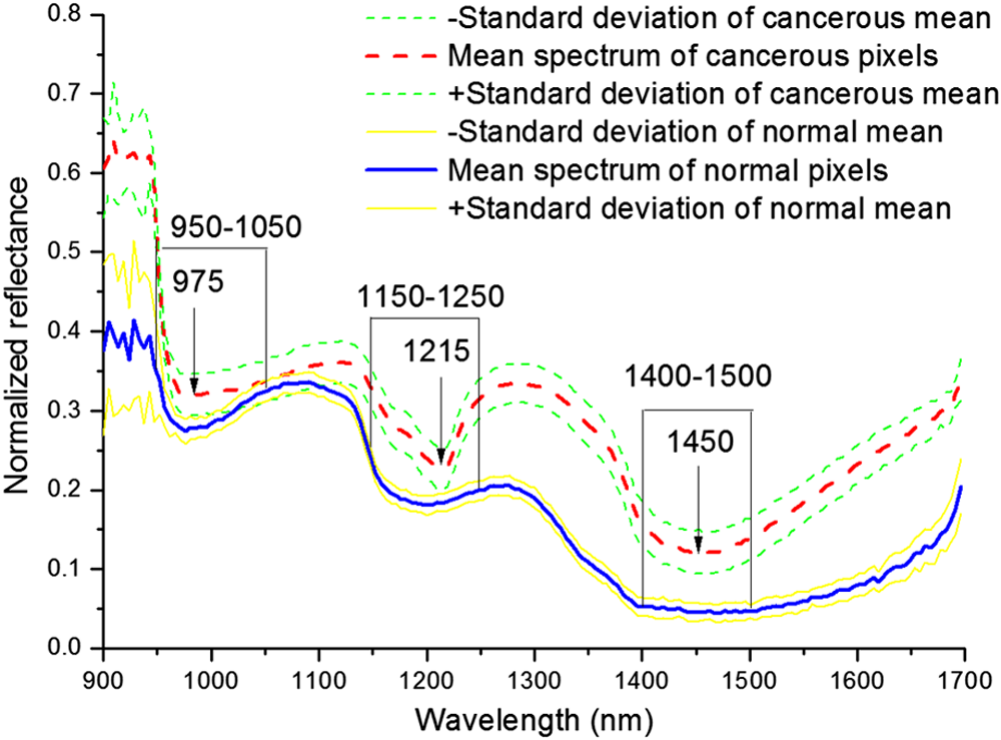
Mean spectrum (red dash line) and standard deviation (green dash lines) of cancerous pixels, mean spectrum (blue solid line) and standard deviation (yellow solid lines) of normal pixels.

Figure 2 shows mean spectra and standard deviation of the reflectance of the pixel from the normal and cancerous regions. Red solid and blue lines represent the mean spectra of cancerous and normal pixels, respectively. The rest are the standard deviations that are nearly constant during the whole spectral range, which suggests the spectra are highly reproducible.

Comparing the mean spectra, a distinctly observed feature useful for recognition of cancerous and normal tissues is that the reflectance level of cancerous tissue is higher than those of normal tissue during the whole wavelength region. This is probably due to the physical surface properties, such as color, shape, and texture. A similar result was observed by Liu et al.^19^ detecting tongue cancer in medical hyperspectral images. Cancerous and normal tissues show different spectral shapes according to their different chemical structure and composition. The most prominent absorption bands occurring in the NIR region are related to overtones and combinations of fundamental vibrations of C-H, N-H, O-H, and S-H functional groups.^20^

According to Fig. 2, the spectral difference can be clearly identified in three regions (950 to 1050 nm, 1150 to 1250 nm, and 1400 to 1500 nm). By inspecting cancerous mean spectra, it was found that the major absorption peaks were observed around 975, 1215, and 1450 nm. The most intensive absorption band around 1450 nm is related to O-H stretching first overtone.^21^ The moderate absorption band around 1215 nm is attributed to C-H stretching second overtone.^22^ The weak absorption band around 975 nm is assigned to O-H stretching second overtone.^21^ The whole spectrum is a mixture of the spectral signature of many tissue components, including water, lipids, proteins, and carbohydrates.^23^^,^^24^

Yi et al. have investigated the application of near-infrared (NIR) spectroscopy equipped with a fiber-optic probe for differentiation gastric cancer. Major spectral differences were observed in the C-H stretching first overtone, combination band, and second overtone regions.^25^ By the use of unsupervised pattern recognition, all spectra were classified into cancerous and normal tissue groups with high accuracy. Similar discrepancy of NIR spectrum for the diagnosis of pancreatic and colorectal cancer has been reported by Kondepati et al.^23^^,^^24^ The similarity indicates the coherence of carcinoma differentiation by NIR. These results show the high discriminating power of the NIR spectrum extraction from hyperspectral image in the identification of cancerous and normal tissue spectral attributes.

### 3.3 PCA and Optimal Wavelengths

**Fig. 3:**
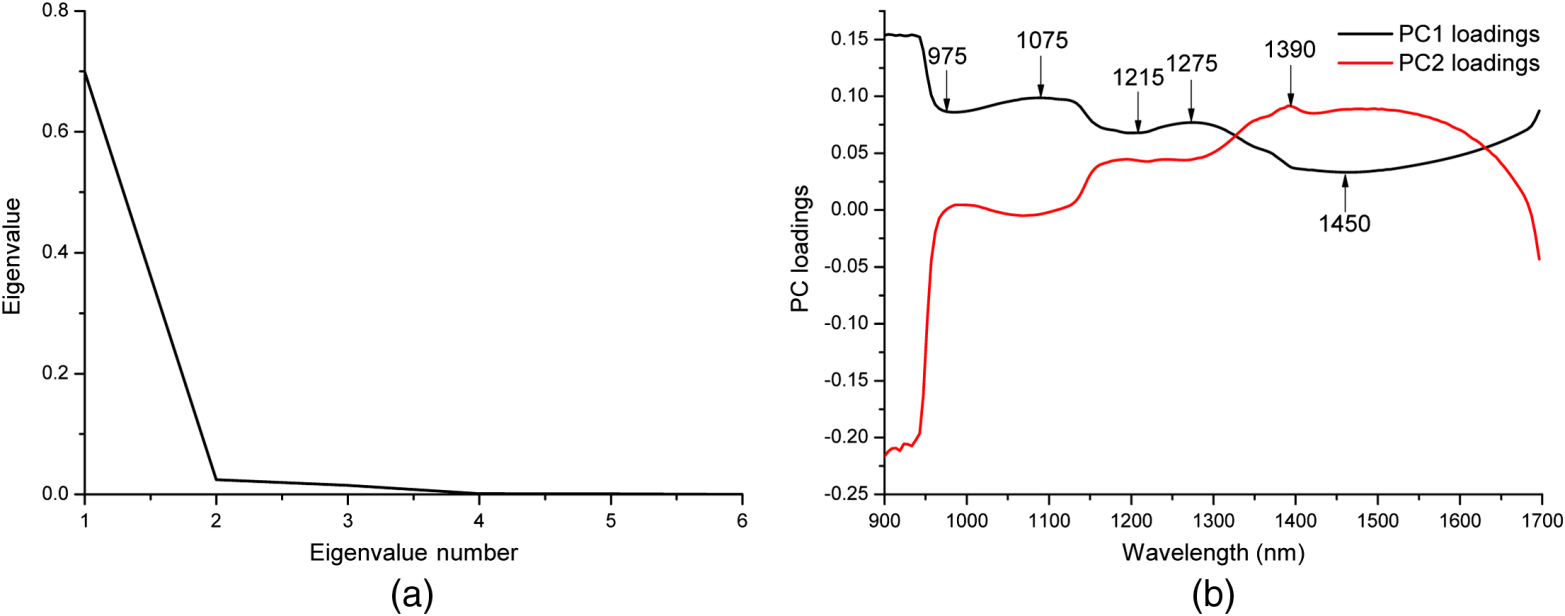
PCA and optimal wavelengths: (a) PC eigenvalue plot and (b) a total of six wavelengths (i.e., 975, 1075, 1215, 1275, 1390, and 1450 nm) selected as optimal wavelengths.

Figure 3(a) shows the PC eigenvalue plot. The horizontal axis represents the eigenvalue number and the vertical axis represents the eigenvalue. The number of PC can equal the score of bands in the original images; however, only the first few PC contain the majority of uncorrelated information.^14^ The first PC contains the largest percentage of data variance, the second PC contains the second largest percentage of data variance, and so on. The last PC appears noisy because it contains very little variance, much of which is due to the noise in the original spectral data. The relative weights of PCs were calculated by dividing their eigenvalues in the eigenvector. PC1 and PC2 explain 94.22% and 3.28% of original variance, respectively.

From the plot of wavelengths versus the loadings of the first and second PCs, it was decided to select those wavelengths situated at the maxima or minima at each plot, as shown in Fig. 3(b). Five wavelengths (i.e., 975, 1075, 1215, 1275, and 1450 nm) were selected from PC1 and only one wavelength (i.e., 1390 nm) was selected from PC2. A total of six wavelengths were then extracted as optimal wavelengths that can be used to discriminate cancer from normal.^18^ These optimal wavelengths not only reflect the physical/chemical information but also maintain the robust diagnosis and classification efficiency.^26^

In this case, PCA was employed to reduce the dimensionality of the datasets from 168 spectral dimensions to only six dimensions. Moreover, these optimal wavelengths, which later may be implemented in a real-time multispectral imaging system, will decrease image acquisition and processing time significantly.^18^

### 3.4 SAM and Supervised Classification

**Fig. 4:**
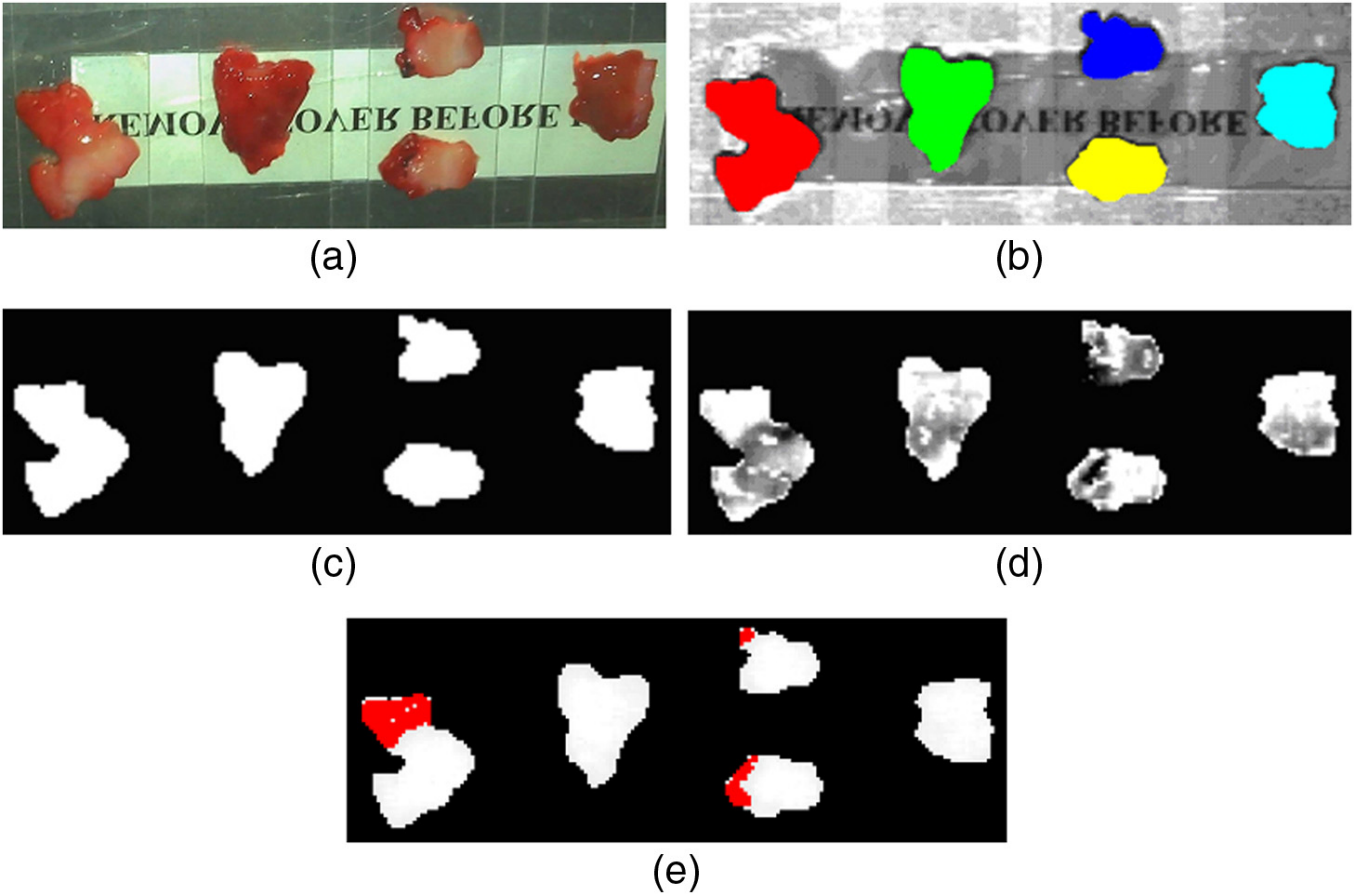
Five representative images of gastric tissues for model training, comprising (a) conventional RGB images, (b) ROI images selected by ENVI ROI tool, (c) binary images, (d) masked images, and (e) gastric cancer diagnosis result by training SAM.

Figure 4 shows some selected images, which comprise the conventional RGB images [Fig. 4(a)], ROI images [Fig. 4(b)], binary images [Fig. 4(c)], masked images [Fig. 4(d)], and gastric cancer diagnosis by training SAM [Fig. 4(e)].

Figure 4(a) shows the conventional RGB image of gastric tissues with impalpable distinction in color. It is not a small challenge for clinicians to distinguish where it is normal and abnormal using conventional reflectance endoscope.^4^ Figures 4(b)–4(d) show the processing results of hyperspectral images for spectral extract and analysis. Figure 4(e) shows the diagnosis result image with training SAM. The red regions are cancerous pixels and the rest are normal ones in Fig. 4(e). The classification result is clear and obvious, which offers great assistance to clinicians.

**Fig. 5:**
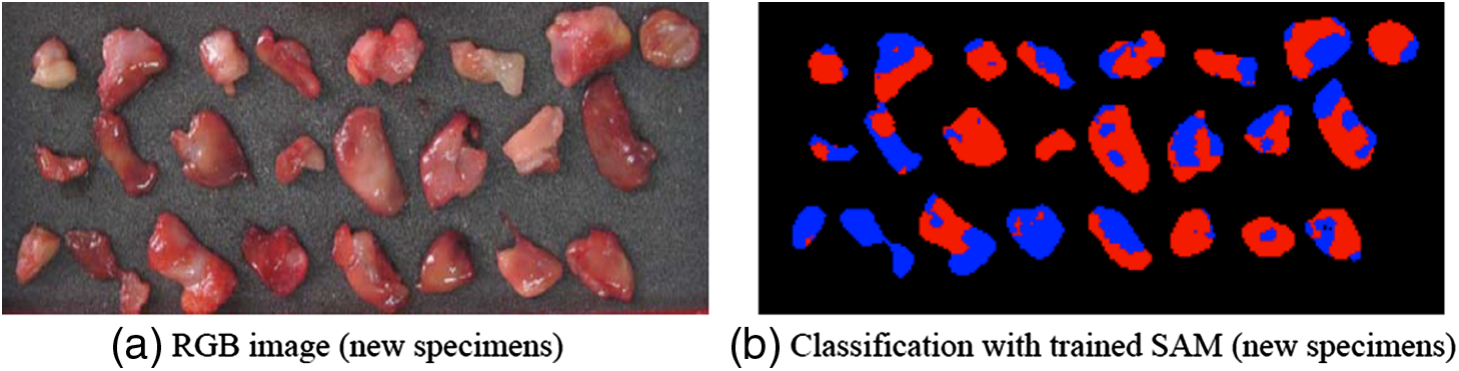
Representative images of gastric tissues by trained model: (a) conventional RGB images and (b) classification map (cancerous regions colored with red and the normal ones colored with blue) with trained SAM for new specimens.

Figure 5 shows the conventional RGB image [Fig. 5(a)] and gastric cancer diagnosis result by trained SAM [Fig. 5(b)] for 24 new samples. In Fig. 5(b), cancerous regions were colored with red and the normal ones were colored with blue. For assessment of the effect of HSI technique, histopathological results served as the “gold standard.” Accuracy is given by the ratio of (TP + TN)/(TP + FP + TN + FN), where TP and FN are the number of the true positive (cancer) and false negative results and TN and FP are the number of true negative (normal) and false positive results, respectively.^27^ While comparing the classification results in Fig. 5(b) with H&E results, the accuracy is up to 90%, which testifies SAM classification algorithms are relatively effective for cancer diagnosis with HSI.^4^ The diagnostic effect with SAM is better than other algorithms’ according to the accuracy,^7^^,^^8^ because SAM is insensitive to illumination and albedo effects, spectral angle will be relatively insensitive to changes in pixel illumination, increasing or decreasing illumination does not change the direction of the vector, only its magnitude.^28^

On one hand, using NIR (900 to 1700 nm), including tissue window^29^ as working wavelengths instead of visible-light region (400 to 800 nm),^6^^–^^8^ will improve the tissue penetration depth and get more diagnostic information,^5^ which could detect lesions in the submucosa of the tissue. On the other hand, using the six optimal wavelengths as input endmember have tremendously decreased the spectral processing time nearly up to real-time requirement with relatively high accuracy. Furthermore, this work and other studies^6^^–^^10^ using HSI without time-consuming stains show that HSI technology may provide a new tool for histological analysis, which could improve both morphometric and biochemical analysis at the same time.

## 4 Conclusions

Hyperspectral images of gastric tissues were captured using an NIR-HSI system. Characteristic spectra of cancerous and normal tissues were extracted. Major spectral differences were observed around 975, 1215, and 1450 nm. Moreover, PCA was used to compress the data dimensions and select the optimal wavelengths. A total of six wavelengths (i.e., 975, 1075, 1215, 1275, 1390, and 1450 nm) were selected as optimal wavelengths that can be used to discriminate between cancer and normal. Furthermore, SAM was utilized to classify cancerous and normal tissues, and the accuracy is up to 90% according to H&E results. These results further suggest that NIR-HSI has the potential for gastric cancer diagnosis and classification with PCA and SAM. Since sample excision and processing are not required for optical diagnosis, a more complete examination *in situ* of the ROI can be achieved than with excision biopsy or cytology. The most important application for optical diagnosis is the possible use for real-time guidance during surgical intervention and treatment.

## References

[1] Siegel R. L. Miller K. D. Jemal A., “Cancer statistics, 2019,” CA Cancer J. Clin. 69(1), 7–34 (2019). CAMCAM 0007-9235 10.3322/caac.21551 30620402

[2] Leung W. K. et al., “Screening for gastric cancer in Asia: current evidence and practice,” Lancet Oncol. 9(3), 279–287 (2008). LOANBN 1470-2045 10.1016/S1470-2045(08)70072-X 18308253

[3] Lu G. Fei B., “Medical hyperspectral imaging: a review,” J. Biomed. Opt. 19(1), 010901 (2014). JBOPFO 1083-3668 10.1117/1.JBO.19.1.010901 PMC3895860 24441941

[4] Halicek M. et al., “*In-vivo* and *ex-vivo* tissue analysis through hyperspectral imaging techniques: revealing the invisible features of cancer,” Cancers 11(6), 756 (2019). 10.3390/cancers11060756 PMC6627361 31151223

[5] Li Q. et al., “Review of spectral imaging technology in biomedical engineering: achievements and challenges,” J. Biomed. Opt. 18(10), 100901 (2013). JBOPFO 1083-3668 10.1117/1.JBO.18.10.100901 24114019

[6] Akbari H. et al., “Cancer detection using infrared hyperspectral imaging,” Cancer Sci. 102(4), 852–857 (2011). 10.1111/j.1349-7006.2011.01849.x 21205093 PMC11158586

[r7] Kiyotoki S. et al., “New method for detection of gastric cancer by hyperspectral imaging: a pilot study,” J. Biomed. Opt. 18(2), 026010 (2013). JBOPFO 1083-3668 10.1117/1.JBO.18.2.026010 23389679

[r8] Goto A. et al., “Use of hyperspectral imaging technology to develop a diagnostic support system for gastric cancer,” J. Biomed. Opt. 20(1), 016017 (2015). JBOPFO 1083-3668 10.1117/1.JBO.20.1.016017 25604546

[r9] Baltussen E. J. M. et al., “Hyperspectral imaging for tissue classification, a way toward smart laparoscopic colorectal surgery,” J. Biomed. Opt. 24(1), 016002 (2019). JBOPFO 1083-3668 10.1117/1.JBO.24.1.016002 PMC6985687 30701726

[10] Han Z. et al., “*In vivo* use of hyperspectral imaging to develop a noncontact endoscopic diagnosis support system for malignant colorectal tumors,” J. Biomed. Opt. 21(1), 016001 (2016). JBOPFO 1083-3668 10.1117/1.JBO.21.1.016001 26747475

[11] Yi W. et al., “Gastric cancer target detection using near-infrared hyperspectral imaging with chemometrics,” Proc. SPIE 9230, 92301V (2014). PSISDG 0277-786X 10.1117/12.2068821

[12] Old O. et al., “Vibrational spectroscopy for cancer diagnostics,” Anal. Methods 6(12), 3901–3917 (2014). AMNEGX 1759-9679 10.1039/c3ay42235f

[13] Kendall C. et al., “Vibrational spectroscopy: a clinical tool for cancer diagnostics,” Analyst 134(6), 1029–1045 (2009). ANLYAG 0365-4885 10.1039/b822130h 19475128

[14] Baranowski P. et al., “Detection of early bruises in apples using hyperspectral data and thermal imaging,” J. Food Eng. 110(3), 345–355 (2012). JFOEDH 0260-8774 10.1016/j.jfoodeng.2011.12.038

[15] Honarmand M. Ranjbar H. Shahabpour J., “Application of principal component analysis and spectral angle mapper in the mapping of hydrothermal alteration in the Jebal–Barez Area, Southeastern Iran,” Resour. Geol. 62(2), 119–139 (2012). RSSSDK 0197-7490 10.1111/j.1751-3928.2012.00184.x

[16] Qin J. et al., “Detection of citrus canker using hyperspectral reflectance imaging with spectral information divergence,” J. Food Eng. 93(2), 183–191 (2009). JFOEDH 0260-8774 10.1016/j.jfoodeng.2009.01.014

[17] Teh S. K. et al., “Diagnostic potential of near-infrared Raman spectroscopy in the stomach: differentiating dysplasia from normal tissue,” Br. J. Cancer 98(2), 457–465 (2008). 10.1038/sj.bjc.6604176 18195711 PMC2361456

[18] Kamruzzaman M. et al., “Application of NIR hyperspectral imaging for discrimination of lamb muscles,” J. Food Eng. 104(3), 332–340 (2011). JFOEDH 0260-8774 10.1016/j.jfoodeng.2010.12.024

[19] Liu Z. Wang H. Li Q., “Tongue tumor detection in medical hyperspectral images,” Sensors 12(1), 162–174 (2012). SNSRES 0746-9462 10.3390/s120100162 22368462 PMC3279206

[20] Garini Y. Young I. T. Mcnamara G., “Spectral imaging: principles and applications,” Cytom. Part A 69(8), 735–747 (2006).1552-4922 10.1002/cyto.a.20311 16969819

[21] Prieto N. et al., “Ability of near infrared reflectance spectroscopy (NIRS) to estimate physical parameters of adult steers (oxen) and young cattle meat samples,” Meat Sci. 79(4), 692–699 (2008). MESCDN 0309-1740 10.1016/j.meatsci.2007.10.035 22063031

[22] Andres S. et al., “The use of visible and near infrared reflectance spectroscopy to predict beef M. longissimus thoracis et lumborum quality attributes,” Meat Sci. 78(3), 217–224 (2008). MESCDN 0309-1740 10.1016/j.meatsci.2007.06.019 22062273

[23] Kondepati V. R. et al., “Application of near-infrared spectroscopy for the diagnosis of colorectal cancer in resected human tissue specimens,” Vib. Spectrosc. 44(2), 236–242 (2007). VISPEK 0924-2031 10.1016/j.vibspec.2006.12.001

[24] Kondepati V. R. et al., “CH-overtone regions as diagnostic markers for near-infrared spectroscopic diagnosis of primary cancers in human pancreas and colorectal tissue,” Anal. Bioanal. Chem. 387(5), 1633–1641 (2007). ABCNBP 1618-2642 10.1007/s00216-006-0960-x 17205263

[25] Yi W. et al., “Gastric cancer differentiation using Fourier transform near-infrared spectroscopy with unsupervised pattern recognition,” Spectrochim. Acta A 101, 127–131 (2013). 10.1016/j.saa.2012.09.037 23099170

[26] Liu Y. et al., “Development of simple algorithms for the detection of fecal contaminants on apples from visible/near infrared hyperspectral reflectance imaging,” J. Food Eng. 81(2), 412–418 (2007). JFOEDH 0260-8774 10.1016/j.jfoodeng.2006.11.018

[27] Akbari H. et al., “Hyperspectral imaging and quantitative analysis for prostate cancer detection,” J. Biomed. Opt. 17(7), 076005 (2012). JBOPFO 1083-3668 10.1117/1.JBO.17.7.076005 22894488 PMC3608529

[28] Petropoulos G. P. et al., “A comparison of spectral angle mapper and artificial neural network classifiers combined with LandSat TM imagery analysis for obtaining burnt area mapping,” Sensors 10(3), 1967–1985 (2010). SNSRES 0746-9462 10.3390/s100301967 22294909 PMC3264462

[29] Pellicer A. del Carmen Bravo M., “Near-infrared spectroscopy: a methodology-focused review,” Semin. Fetal Neonatal. Med. 16(1), 42–49 (2011). 10.1016/j.siny.2010.05.003 20580625

